# *Brucella* Seropositivity and Associated Risk Factors in Pastoral Livestock System in Northeastern Ethiopia

**DOI:** 10.3390/vetsci11120620

**Published:** 2024-12-03

**Authors:** Berhanu Sibhat, Haileeyesus Adamu, Teferi Benti, Getachew Tuli, Kassahun Asmare, Johanna F. Lindahl, Ulf Magnusson, Tesfaye Sisay Tessema

**Affiliations:** 1 Institute of Biotechnology, Addis Ababa University, Addis Ababa P.O. Box 1176, Ethiopia; tesfaye.sisayt@aau.edu.et; 2 College of Veterinary Medicine, Haramaya University, Dire Dawa P.O. Box 138, Ethiopia; 3 Division of Reproduction, Department of Clinical Sciences, Faculty of Veterinary Medicine and Animal Science, Swedish University of Agricultural Sciences, P.O. Box 7054, 750 07 Uppsala, Sweden; johanna.lindahl@slu.se (J.F.L.); ulf.magnusson@slu.se (U.M.); 4 Animal Health Institute, Sebeta P.O. Box 04, Ethiopia; teferibenti58@gmail.com (T.B.); getechewtuli@yahoo.com (G.T.); 5 Faculty of Veterinary Medicine, Hawassa University, Hawassa P.O. Box 05, Ethiopia; kassahun@hu.edu.et; 6 Department of Animal Health and Antibiotic Strategies, Swedish Veterinary Agency, 751 89 Uppsala, Sweden

**Keywords:** brucellosis, cattle, camel, goats, pastoral systems, public awareness, sheep

## Abstract

Brucellosis is a common zoonotic disease affecting livestock and humans globally. The disease is endemic in Ethiopian livestock. This study was conducted to estimate *Brucella* seropositivity and identify its risk factors in livestock, and practices that may expose pastoralists to the disease. Data were collected from 2133 animals across 149 households (HHs) in Dubti and Amibara districts, Afar region, Ethiopia. Blood samples from livestock and household data were collected, and interviews were conducted on husbandry and dairy consumption practices. Sera were serially tested using the Rose Bengal test and indirect enzyme-linked immunosorbent assay. The overall seropositivity to anti-*Brucella* antibodies was 8% (95% CI, 6.6–9.2). The antibodies were found in 12.4%, 6.5%, 6%, and 3% of the goats, cattle, camels, and sheep, respectively. Among the HHs, 59.7% had at least one seropositive animal. A mixed-effects logistic regression analysis revealed species and the acquisition of new animals (in cattle and camels), and age and district (in sheep, goats, and cattle) as significant risk factors. Goats, cattle, and camels had significantly higher odds of *Brucella* seropositivity than sheep (*p* < 0.05). Seropositivity was significantly higher (*p* < 0.05) in adults than in young animals, in acquired than in homebred (cattle and camels), and in those with reproductive disorders than those without. Pastoralists lacked knowledge of brucellosis and its modes of transmission, while practices exposing humans and livestock to brucellosis were common. The findings underscore the need for public awareness campaigns and implementation of brucellosis control measures in pastoral systems.

## Simple Summary

Brucellosis is an infectious disease that affects both animals and humans. It is caused by bacteria called *Brucella* and leads to serious reproductive problems in animals, such as late-term abortions and stillbirths. In humans, the disease causes a long-lasting illness characterized by undulating fever, malaise and infections of bones and joints, causing severe pain. People can catch brucellosis through direct contact with infected animal birth or abortion-related fluids or by consuming unpasteurized milk and dairy products. This study looked at the presence of brucellosis among livestock kept by Afar pastoralists in northeastern Ethiopia. Blood samples from goats, sheep, cattle, and camels were tested for exposure to the bacteria. Interviews with livestock owners revealed their practices, including milk consumption and animal care. The results show that 8% of the animals were infected, with goats being the most affected (12.7% positive). Many herders did not know about the disease or how it spread. Risky practices like drinking raw milk, assisting with animal births barehanded, and discarding aborted fetuses in the fields were found to be common, increasing the risk of infection and environmental contamination. The study concludes that brucellosis is widespread in livestock, and that both animals and humans are at risk. It recommends better disease control and education to prevent further spread.

## 1. Introduction

Brucellosis is a disease of livestock and also one of the most common zoonoses globally [1]. In animals, it is characterized by reproductive disorders such as abortion in the last stages of pregnancy, stillbirth, or birth of weak offspring, and epididymitis and orchitis in males [2,3]. In humans, it causes a chronic debilitating disease with non-specific clinical symptoms such as fever, malaise, arthritis, and other symptoms depending on the organs or systems involved [4].

The disease is caused by members of the genus *Brucella* [5]. *Brucella abortus* causes brucellosis primarily in cattle, *Brucella melitensis* in small ruminants, and *Brucella suis* in swine [6]. Camels are mostly infected by *B. melitensis* or *B. abortus* depending on the degree of association with either small ruminants or cattle, respectively [7]. Spill-over of *Brucella* infections from preferential hosts to other non-preferential host species in mixed livestock herds is common [8,9].

Brucellosis remains a significant global public health concern. High-risk areas often have socio-economic and environmental conditions that facilitate the spread of the disease, especially in communities where livestock plays a vital role in food and economic security. The disease is endemic in several countries in sub-Saharan Africa, Asia, South and Central America, and countries along the Mediterranean Sea [10,11].

A collation of previous serological studies using meta-analysis showed that brucellosis is endemic in livestock in Ethiopia with pooled seropositivity of 4% in goats, 3% each in sheep and camel, and 2.6% in cattle [12]. The distribution, however, is not consistent across the country, with greatly varying seropositivity ranging from 0 to 35% in goats [13,14,15], 0 to 17% in sheep [15,16], 0 to 19% in cattle [17,18,19] and 0 to 14% in camels [19,20] at individual animal level. Afar region, northeastern Ethiopia, and the pastoral livestock production systems had the highest seropositivity to *Brucella* compared with other regions of the country and production systems [21].

There are few studies on all livestock species kept by herders in the same HHs globally. Such studies may help in identifying the relative importance of livestock species in brucellosis epidemiology in the area. The objectives of this study were to estimate the prevalence of seropositivity to *Brucella* infection and to identify risk factors associated with livestock kept by HHs in pastoral production systems of the Afar region in northeastern Ethiopia. This study also aimed to identify knowledge gaps and practices in such production systems that might increase public health risks from exposure to *Brucella*.

## 2. Materials and Methods

### 2.1. Study Area

**Figure 1:**
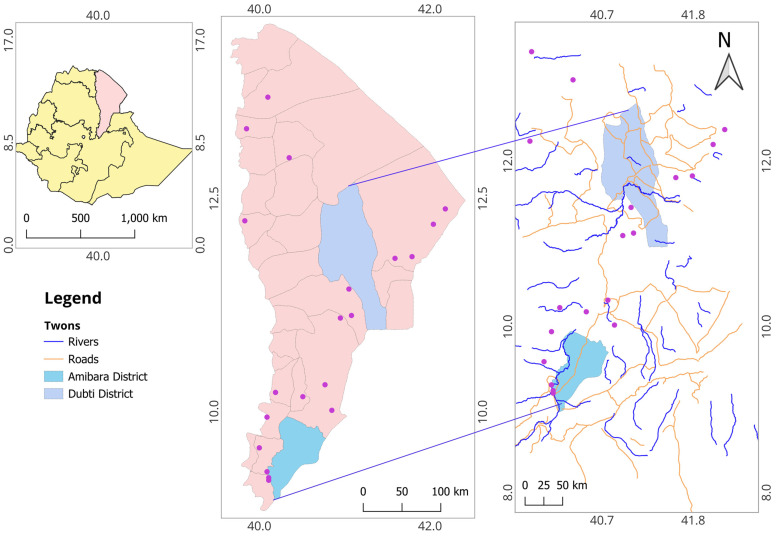
Map of Afar region with Amibara and Dubti districts (far left: map of Ethiopia with Afar region marked in pink), where livestock blood samples were collected for *Brucella* serology.

The study was conducted in the Amibara and Dubti districts of the Afar regional state between February and June 2021. Amibara and Dubti are located 260 km and 607 km northeast of Addis Ababa, respectively, with average altitudes of 740 and 503 m above sea level, situated in the Mid and Lower Awash Valley of Afar region, respectively (Figure 1) [22]. The districts are each home to approximately 65,000 inhabitants [23]. Agro-ecologically, Amibara is semi-arid with a temperature ranging from 25 to 35 °C and an average annual rainfall of 530 mm, while the corresponding figures for Dubti are 18 to 35 °C and 214 mm. Most rains occur from July through September while the driest months are May and June [24]. Transhumance pastoralism is the major livestock production system in both districts, where cattle, camel, goats, and sheep are raised [25]. Livestock are kept primarily for milk and meat for self-sufficiency and local sale.

### 2.2. Study Animals

The study included indigenous cattle, sheep, goats, and camels kept by the Afar pastoral communities of the two districts. Similar breeds of Afar goats, Afar sheep, and Afar cattle, named after the local Afar people, were kept in both districts. In the Afar region, two camel ecotypes are recognized: the Amibara ecotype, common in Amibara and adjacent districts of southern Afar where the climate is semi-arid, and the Mile ecotype, found in central areas of Afar (including Mile, Chifra, and Dubti districts), where the climate is more arid [26]. Only animals above six months of age were included in the sampling of all livestock species. The term ‘small ruminant’ includes sheep and goats.

### 2.3. Study Design and Sampling Methodology

The study was a cross-sectional study that included questionnaire-based interviews of pastoralists in the two districts holding goats, sheep, cattle and camels, and blood sampling of the livestock. Districts were purposively selected based on their accessibility, abundance of livestock, and relative security during sampling time. Within the two districts, pastoral villages were selected in consultation with district livestock authorities and based on transportation accessibility and availability of livestock near permanent settlement areas during planned sampling. Within the villages, HHs were selected randomly from a list of pastoralists prepared by the animal health workers at the villages and the district veterinarian, together with community elders.

Animals of all livestock species kept by the selected HHs were selected for blood sampling using systematic random selection, where the *n*th animal was selected when the number of animals was greater than 50. If the number of animals was less than 50 and kept in separate household-level enclosures (especially in small ruminants), simple random sampling using the lottery method was applied. In cases where animals were kept together at a village level (common for cattle and camels, especially in the Amibara district), lists of animal names provided by the owners (the livestock did not have ear tags or any form of identity numbers), were used as sampling frames to select the animals randomly using the lottery method.

### 2.4. Sample Size Determination

The sample size was calculated using EpiInfo^TM^ version 7.2.2.16 (Centers for Disease Control and Prevention, USA). Prevalence estimates for each livestock species from previous studies, 95% confidence level, 5% absolute precision, design effect value of 2.0, and 70 HHs as clusters were used as inputs for each of the districts. Expected sero-prevalence of 5% [21], 7%, 13.6% [27], and 5.4% [28] were used as inputs for pastoral cattle, sheep, goats, and camels, respectively. Accordingly, it was calculated that a minimum of 1050 animals were required to be sampled from each district. This represented six goats and three animals each for sheep, cattle, and camels from selected HHs, depending on the species kept. In total, 1063 and 1070 animals were sampled from Dubti and Amibara districts, respectively. Species-wise, 844 goats (786 females and 58 males), 434 sheep (409 females and 25 males), 429 cattle (398 females and 31 males), and 426 camels (412 females and 14 males) were included. Altogether, 2133 animals composed of 2005 females and 128 males, were sampled from both districts.

### 2.5. Sample and Data Collection

Blood samples were collected from the jugular vein with sterile needles and plain vacutainer tubes (Becton, Dickinson and Company, Gurgaon, Haryana, India). The samples were left to stand overnight at an ambient temperature (20–25 °C) for separation into serum and clot. Those that failed to produce a clear separation between the two phases were centrifuged at 2000× *g* for 10 min to separate the sera manually from the clots. Within 12–24 h, the sera were transferred to duly labeled sterile screw-capped cryovial tubes (Thermo Scientific, Waltham, MA, USA) using sterile disposable Pasteur pipettes (Chemglass Life Sciences, Somerset, NJ, USA) and stored at −20 °C until further analysis.

Data on individual animal characteristics were collected by filling out a questionnaire during the bleeding of each animal using a format prepared for this purpose (Supplementary Table S1). No written records about individual animals or herds were available at any of the visited HHs. Therefore, all of the information obtained was dependent on the recollections of the individual livestock owners.

A questionnaire was used to obtain data on individual HH characteristics and livestock management practices (Supplementary Table S2). The data included the gender and age of the household head, level of formal education, type of livestock species kept, herd size, contacts between the different livestock species within the HH and at the village level, and contact with wildlife at pasture, watering points, and night-time housing places. Furthermore, data on livestock management practices were also collected, including data related to the ownership and management of pasture, ownership of watering resources, type of animal breeding methods (artificial insemination, natural controlled breeding with known sire, or natural uncontrolled breeding), presence of designated parturition pen and common places where delivery occurred, delivery/abortion waste management, and the presence or absence of practices of isolating aborting animals. All data from the questionnaires were entered into an Excel spreadsheet and later imported into Stata 14.2 (Stata Corp., College Station, TX, USA) for analysis.

Information on household milk consumption habits and on preferences for milk from various livestock species kept at the household and for post-milking processes (boiling, processing to other local milk products) were collected. Other data collected include the knowledge and practices of the pastoralists potentially related to *Brucella* exposure. These included whether they milk and use milk from aborting animals, receive assistance during delivery/abortion, use personal protection while doing so (use of gloves and hand washing practices afterward), and whether the respondent knew of brucellosis and its modes of transmission between animals and between animals and humans.

Observation checklists for animal housing included floor type, the presence of separate housing for the different livestock and different age groups, cleanliness (based on the presence or absence of accumulated livestock dung), the presence of drainage facilities, and the presence of private farm waste dumping sites near the farms.

### 2.6. Laboratory Analyses

Serum samples were initially screened at the Animal Health Institute (AHI), Sebeta, Ethiopia, using the Rose Bengal plate test (RBPT) (Source of Rose Bengal antigen: Cenegenics Corp., Morganville, NJ, USA) according to the procedures described by the World Organization for Animal Health [29] for the different animal species.

Confirmation of the RBPT-positive samples for anti-*Brucella* antibodies was made using an ID Screen^®^ brucellosis serum indirect enzyme-linked immunosorbent assay (iELISA) multi-species kit (Innovative Diagnostics, Grabels, France) according to the procedures recommended by the manufacturer.

Briefly, RBPT-positive test sera from livestock (diluted to 1:20) and control sera provided with the test kit were dispensed into *Brucella* lipopolysaccharide (LPS)-coated 96-well ELISA plates. The plates were then incubated at room temperature (20–25 °C) for 45 min. After washing, anti-multi-species-immunoglobulin G (IgG)-horseradish peroxidase (HRP) conjugate was added to each well and incubated at room temperature for 15 min. This conjugate is designed to detect anti-*Brucella* spp. antibodies in sera of cattle, sheep, goats, and swine. The excess conjugate was then washed out, a color-producing substrate (tetramethylbenzidine) was added and the plates were kept at room temperature in a dark place for 15 min. Finally, a stop solution was added to stop the reaction, and the plates were read using an ELISA microplate reader (Multiskan FC, Thermo Scientific, Waltham, MA, USA) at the optical density (OD) of 450 nm. The sample positive ratio (S/P%) was calculated based on the following equation provided in the test manual.

$$\frac{S}{P}\%=\frac{OD_{sample}-OD_{NC}}{OD_{PC}-OD_{NC}}\times 100$$

where *OD_sample_* is the optical density of the test sample, *OD_NC_* is the optical density of the negative control, and *OD_PC_* is the optical density of the positive control.

Samples were classified as negative when the S/P% was 110% or less, doubtful when 110% < S/P% < 120%, and positive when S/P% was 120% and above. According to the manufacturer, the diagnostic sensitivity and specificity of the ID Screen^®^ brucellosis ELISA were 100% and 99.7%, respectively. Furthermore, a meta-analysis of brucellosis diagnostic tests shows that the sensitivity of an iELISA using LPS was 98.3 (95% confidence interval (CI) 97.1–99.0%), and the specificity was 99.7% (95% CI 99.5–99.8%) [30].

A sample was considered *Brucella*-seropositive if it was found to be positive first by RBPT and then again by iELISA. As livestock in Ethiopia had never been vaccinated for *Brucella* spp., a positive antibody test is considered as exposure to *Brucella* spp.

### 2.7. Data Analyses

Potential risk factors for an animal to be *Brucella* seropositive, and the possible association between *Brucella* serostatus and reproductive problems (abortion, retained fetal membranes and stillbirth as outcome variables) were assessed using multivariable mixed effects and univariable logistic regression, respectively. Stata SE 14.2 (Stata Corp., College Station, TX, USA) was used for data analyses. A predictor was selected for further analysis based on the prevailing biological plausibility [31]. The collinearity between independent variables was verified in a cross-tabulation using Goodman and Kruskal’s gamma statistic, with a cut point of gamma at ≥+0.6 or ≤−0.6. All non-collinear predictors with *p*-value < 0.3 in univariable analyses were included in the multivariable mixed effects logistic regression model using the *melogit* command in Stata, with the default integration method and points (i.e., mean-variance adaptive Gauss–Hermite quadrature and 7, respectively) and while considering HH and village as random effects to control for clustering. The final model was optimized using the backward exclusion of non-significant variables (*p* > 0.05). A *p*-value less than or equal to 0.05 was considered for statistical significance. Details of the analyzed variables and their categories were described in Supplementary Material Table S2.

## 3. Results

### 3.1. Household Characteristics and Livestock Management Practices

Households sampled in the Amibara district kept a significantly higher (*p* < 0.05) mean number of goats, sheep and cattle than the HHs in the Dubti district. The mean number of camels, however, was not significantly different (*p* > 0.05) between the HHs in both districts (Supplementary Table S3).

According to the responses of the pastoralists, pasture and water resources were shared communal properties. All 149 respondents stated that livestock from the household had frequent contact with livestock from other HHs in the village at pasture and watering sites. They also mentioned that livestock came into contact with livestock from other villages at pasture and watering sites. Additionally, contact with wildlife while grazing in the pasture was mentioned by all of the respondents. In both districts, livestock breeding was uncontrolled, and mating occurred naturally at the pasture or in livestock enclosures with any available male animal. None of the HHs had separate pens for delivery. Every HH reported that livestock delivery occurred both at night-time enclosures and at pasture. Pastoralists helped animals during delivery and abortion with bare hands. All of the respondents stated that, after delivery, wastes such as placenta and/or abortion materials were discarded in open fields. They also indicated that animals experiencing abortions were not isolated from the herd after the event.

### 3.2. Knowledge of Brucellosis and Household Milk Consumption Habits

None of the pastoralists knew that brucellosis is a zoonotic disease. They reported having never boiled milk from livestock. All of the HHs also reported consumption of raw milk from all livestock species and used milk from animals that had aborted.

### 3.3. Observations on Animal Housing

Night-time housing facilities for animals were simple fences made of thorny bushes, or wire mesh or plastic meshes when available. In both districts, all of the 138 (92.6%) individual HHs that had both sheep and goats kept them together in common enclosures. The enclosures were meant to separate them from other livestock and protect them from predators. Suckling kids and lambs, as well as calves, were all kept in separate enclosures. Cattle were kept in separate fenced enclosures at all HHs in the Dubti district. In the Amibara district, however, cattle above the age of one year were all communally kept at the center of the villages. Similarly, camels were also kept together close to the villages in both districts. In half of the villages (50%) in Amibara, overnight mixing of camels with cattle and donkeys was observed.

All of the enclosures had earthen floors with no drainage facilities. They were not frequently cleaned as fresh and dried fecal materials were observed piled up in the enclosures. While the enclosures were not frequently cleaned, common dump sites, however, existed at villages in Dubti but not in Amibara.

### 3.4. Sero-Prevalence of Brucella

A total of 2133 animals were sampled from 149 HHs. The overall sero-prevalence of brucellosis in the two districts was 8.0% (95% CI, 6.6–9.2) at the animal level and 59.7% (95% CI, 51.6–67.4) at the household level. Both the overall and household level sero-prevalences were significantly higher in Amibara compared with the Dubti district (*p* < 0.001), (Table 1).

Among all of the livestock species sampled, livestock in Amibara district had significantly higher (*p* < 0.05) *Brucella* seropositivity than the corresponding livestock species in Dubti, except for cattle, where no such differences (*p* > 0.05) were observed (Table 2).

Previous history of reproductive disorders in livestock in the two districts of Afar such as abortion, stillbirth and retained fetal membranes were significantly associated with *Brucella* seropositivity (*p* < 0.001). A breakdown of the data into the various livestock species was found to show that associations were found for small ruminants and cattle (*p* < 0.05) but not for camels (*p* > 0.05) (Table 3).

### 3.5. Risk Factors for Brucella Seropositivity

Among all livestock species, district, species, age, herd size and origin of the animals were identified as significant risk factors for *Brucella* seropositivity in the multivariable mixed effects logistic regression model (Table 4). With regards to species, goats, cattle and camels had greater odds of seropositivity than sheep. Older animals, animals in larger herds and acquired animals had significantly greater odds of being *Brucella* seropositive. Clustering of cases was observed at the HH level.

The final multivariable mixed effects logistic regression model for *Brucella* seropositivity in sheep and goats identified districts, species difference, age and herd size as significant predictors (*p* < 0.05). Clustering was observed both at HH and village levels (Table 5).

The multivariable model predicted the introduction of acquired animals into the HHs as the only significant factor (*p* = 0.049) for *Brucella* seropositivity in cattle at an individual animal level. For camels, district and acquiring animals were the two predictors identified. Clustering was negligible at the village level for both cattle and camel *Brucella* seropositivity. However, it was considerable at HH level in camels (Table 6).

Seropositive animals were found in all of the sampled villages. The seropositivity ranges from 2.6–9.2% in villages in Dubti and from 8.2–11.8% in villages in Amibara district at an individual animal level (Table S5).

### 3.6. Sero-Prevalence at the Household Level

Similar to the individual animal level sero-prevalence, HH level sero-prevalence was the highest in goat-keeping HHs. Among all of the HHs, 46% of goats, 18% of cattle, 15.5% of camel and 10% of sheep-keeping HHs had at least one seropositive animal. Among the seropositive HHs, 73%, 29.2%, 24.7% and 14.6% had seropositive goat, cattle, camel or sheep, respectively (Table S4).

At the HH level, the seropositivity ranges from 32.1–66.7% and 70.6–77.3% in Dubti and Amibara districts, respectively. Some of the villages in Amibara had significantly higher (*p* < 0.05) animal-level seropositivity than villages in Dubti district (Table S5).

At HH level, multivariable mixed effects logistic regression analysis predicted district [OR = 3.7 (95% CI, 1.8–7.7)] as the only significant (*p* < 0.05) putative risk factor. Other factors, including the owner’s age, gender and education level, were not significant (*p* < 0.05) (Table S6).

## 4. Discussion

In this study, none of the pastoralists knew that brucellosis is a zoonotic disease and how it is transmitted. All of them were involved in risky livestock husbandry practices such as unprotected delivery assistance, disposal of parturition wastes such as placenta and aborted fetuses into open fields, and consumption of raw milk, including from animals with a recent history of abortion. This finding agrees with previous reports from the Afar region, in which 96–100% of the pastoralists were found to have no knowledge of the disease and to engage in similar risky practices [32,33]. Similar findings have also been reported from other pastoral areas of Ethiopia [14,19] and pastoral communities in northwest Côte d’Ivoire [34]. In the study area, aborting animals were kept within the herd. Infected animals, especially following abortions, are important sources of infection to animals and humans [2,35]. Additionally, consumption of contaminated unpasteurized dairy products exposes humans to *Brucella* infection [36,37]. Previous findings have indicated *Brucella* seropositivity of 48.3% in Afar and 34.9% among Somali pastoralists residing in areas adjacent to the Afar region [38]. This shows that the risk of *Brucella* infection in humans in the pastoral systems is high and that livestock and public health authorities need to make concerted efforts in raising the public health awareness of the disease and its prevention.

The 8% overall sero-prevalence of brucellosis in livestock in this study corroborates earlier reports of 9% from Afar and 8.6% from the adjacent areas of the Somali region [38]. These results indicate that brucellosis is endemic to pastoral livestock in the region. Our findings and previous systematic reviews [12,21] reveal that the sero-prevalence of brucellosis is higher in the pastoral livestock compared with the sedentary livestock in the mixed crop–livestock production and the urban and peri-urban dairy systems. Similarly, a sero-prevalence of 45.1% in cattle kept under the pastoral system in Nigeria was higher than the sedentary zero-grazing (23.8%) and commercial farm (15.9%) systems [39]. Higher sero-prevalence of brucellosis in the pastoral livestock, as opposed to the mixed crop-livestock production system in Ethiopia and other non-pastoral production systems in Africa, could be associated with livestock mobility and the keeping of large herds in the pastoral systems, which allow close contact between potential sources of infection and a large number of susceptible animals in close confinement [40].

Interestingly, the sero-prevalence of brucellosis in livestock in the Somali pastoral areas of eastern Ethiopia has remained low (0.0 to <3%) [13,16,19], except in the areas bordering Afar region [38]. The difference between the Afar and Somali pastoral livestock is that livestock in Afar migrate across clan borders, bringing together livestock from wider areas of Afar, as opposed to the Somali clan-based system, where only livestock from the same clan congregate, preventing mixing with livestock from different clans [16].

*Brucella* sero-prevalence in livestock was higher in Amibara (10.8%) than in Dubti (5.2%) district, except for cattle. Differences between the districts include herd size and night-time livestock enclosures. Pastoralists in Amibara kept larger herds compared with those in Dubti, which might have increased the risks of infection, especially for small ruminants, which are mostly kept near permanent settlements. Further breakdown of the risk factors along different livestock groups showed that herd size was not significant in cattle and camels. This variation might have occurred due to factors that were not captured by the current study, such as cattle and camel migration patterns, which are differently affected by the climatic conditions of the districts. Despite these differences, anti-*Brucella* antibodies were found in all villages in both districts, but prevalence varied, indicating a patchy distribution of livestock brucellosis ranging from 2.6–11.8%. This mirrors findings from southern Ethiopia [41].

Among the studied livestock, goats with 12.7% had the highest *Brucella* seropositivity. Interestingly, sheep (kept together with goats) with 3% had the lowest seropositivity in both study districts. It is reported that all goat breeds are susceptible to *Brucella* infection while only some breeds of sheep (especially milk breeds) are considered susceptible [36]. In this study, the reason for this difference is unclear, as the results from previous studies in the region provided mixed results. Some of the studies agree with our findings [13,16]. Others, however, did not find these differences [38]. Similar trends, where goats had the highest sero-prevalence among the co-existing livestock species, have been reported from other pastoral systems [42,43]. On the contrary, sheep in Tajikistan were found to be more affected than cohabiting goats [44].

The *Brucella* seropositivity in cattle, found to be 6.5% in this study, conforms with the 5.7% to 6.9% range previously reported for cattle in Afar [38,45]. Several studies have reported a similar seropositivity range of 4–10% from cattle in pastoral and agro-pastoral production systems in Ethiopia [14,41,46]. Generally, cattle in pastoral/agro-pastoral systems in Ethiopia have significantly greater odds of seropositivity than those in the mixed crop–livestock and the urban/peri-urban dairy production systems, as discussed previously [12].

The 5.9% seropositivity of camels reported in this study is within the 5–8% range of previous studies in Afar [28,38]. Much lower sero-prevalence, ranging between 0.0–2.0%, has been reported from pastoral systems in the east and south of Ethiopia [19,47,48]. These differences could be the results of variations in herd management and/or the type of diagnostic tests used in the studies, as described by Sibhat et al. [12]. The serological tests used in the diagnosis of camel brucellosis, such as those used in the current study, have been validated only for use in other livestock, such as cattle and small ruminants, hence their interpretation in camel requires careful consideration. Camels are highly susceptible to brucellosis caused by *B. melitensis* and *B. abortus,* and contract the infections from cohabiting infected small ruminants or cattle, respectively [7,36]. In the study districts, camels shared the environment with small ruminants and cattle. Isolation of *Brucella* from camels in such settings could shed light on the cross-species transmission of the agent.

In serological studies like this one, it is not possible either to differentiate the species of *Brucella* or provide evidence of its cross-transmission among the mixed livestock. However, goats (and *B. melitensis*) likely play a significant role in the epidemiology of brucellosis in the study area, for the following reasons. First, *Brucella melitensis* infection in goats causes the shedding of copious reproductive fluids, which contain the bacteria for at least three months, this is in contrast with sheep and cattle, where shedding mostly occurs only for three weeks following abortions [49]. Second, *Brucella* seropositivity was found to be significantly higher in goats than in other livestock species in the current study. Finally, *B. melitensis* had been previously isolated from vaginal swabs and milk of goats in the study area [50].

*Brucella* seropositivity was found to be associated with reproductive disorders like abortion and stillbirth in small ruminants and cattle, but not camels. This agrees with Megersa et al. [51], who found higher *Brucella* seropositivity in goats and cattle with abortions in southern Ethiopia. Similar findings have been reported in Ethiopia [41], Bangladesh [52], and Pakistan [53]. However, only 65 (17.4%) of the 373 animals with a history of abortions in this study tested seropositive for *Brucella*, suggesting the possible involvement of other causes. In this regard, previous studies have reported seropositivity to *Neospora caninum* and the bovine diarrhea virus in cattle, and *Coxiella burnetii*, *Chlamydia abortus* and *Toxoplasma gondii* in small ruminants, all associated with abortions in Ethiopia [54,55]. Similarly, while no reference was made to reproductive disorders, seropositivity to known abortifacient diseases, including Q-fever and Rift Valley fever, has also been reported from camels in Ethiopia [56]. Therefore, further research is needed to identify the causes of reproductive wastage in pastoral livestock.

One way of introducing brucellosis into farms is through the introduction of infected livestock [57]. In the current study, because of the cross-sectional nature of the design, it is impossible to know whether the acquired animals were exposed to *Brucella* spp. before or after they were introduced into the HHs. However, the odds of being seropositive have been found to be significantly greater in the acquired than in the homebred camels and cattle. Similar trends have been observed in dairy cattle in Ethiopia [58,59]. This might indicate that pastoralists sell female animals after observing reproductive problems, a phenomenon that has also been observed in dairy cattle in central Ethiopia [60].

## 5. Conclusions

Brucellosis is prevalent in livestock in northeastern Ethiopia at individual and household levels. Brucellosis seropositivity was found to be higher in acquired than in homebred cattle and camels, in larger rather than small herds for small ruminants and in female animals with a history of reproductive disorders rather than those without in all livestock, with the exception of camels. Among livestock, the prevalence was highest in goats followed by cattle, camels, and sheep. Awareness of brucellosis among the pastoralists was found to be non-existent, while practices that facilitate the transmission of *Brucella* spp. in livestock and humans were highly prevalent. Our findings suggest the need for the implementation of brucellosis control interventions following the identification of circulating *Brucella* species and the corresponding primary hosts for technical and logistic reasons. Concomitantly, there is a need for public health awareness creation in the Afar region and similar pastoral settings.

## Figures and Tables

**Table 1:** Sero-prevalence of brucellosis in livestock in two districts of Afar, Ethiopia, at the individual animal and household (HH) levels.

District	Individual Animal Level			Household Level		
	No. Sampled	No. Positive	Prevalence (95% CI)	No. HHs Sampled	No. HHs Positive	Prevalence (95% CI)
Amibara	1063	115	10.8 (9.1–12.8) *	71	53	74.6 (63.1–83.5) *
Dubti	1070	56	5.2 (4.0–6.7)	78	36	46.1 (35.3–57.4)
Overall	2133	171	8.0 (6.6–9.2)	149	89	59.7 (51.6–67.4)

* Statistically significant difference within column (*p* < 0.001); CI, confidence interval; HHs, households.

**Table 2:** Individual animal level sero-prevalence of brucellosis in four livestock species in two districts of Afar, Ethiopia.

Species	District	No. Sampled	No. Positive	Percent Seropositive (95% CI)	OR (95% CI)	*p*-Value
Goat	Dubti	420	30	7.1 (4.9–10.0)	Ref.	
	Amibara	424	75	17.7 (14.1–21.7)	2.8 (1.8–4.4)	<0.001
Sheep	Dubti	221	2	0.9 (0.1–2.3)	Ref.	
	Amibara	213	11	5.5 (2.6–9.0)	6.0 (1.3–27.2)	0.021
Cattle	Dubti	216	17	7.9 (4.3–11.5)	Ref.	
	Amibara	213	11	5.2 (2.2–8.1)	0.6 (0.3–1.4)	0.260
Camel	Dubti	213	7	3.2 (0.9–5.7)	Ref.	
	Amibara	213	18	8.4 (4.7–12.2)	2.7 (1.1–6.6)	0.029

CI, confidence interval; OR, odds ratio; Ref., reference.

**Table 3:** Univariable logistic regression analysis of reproductive clinical outcomes with seropositivity to *Brucella* spp. in livestock in the Afar region, Ethiopia.

Reproductive Disorder	Category	No. Examined	No. Positive	Seropositivity (%) (95% CI)	OR (95% CI)	*p*-Value
All livestock species						
Abortion	No	1229	73	5.9 (4.7–7.4)	Ref.	
	Yes	373	65	17.4 (13.9–21.6)	3.3 (2.3–4.8)	<0.001
RFM	No	1556	121	7.8 (6.5–9.2)	Ref.	
	Yes	46	17	36.9 (24.2–51.8)	6.9 (3.7–13.0)	<0.001
Stillbirth	No	1525	119	7.8 (6.6–9.3)	Ref.	
	Yes	77	19	24.7 (16.3–35.6)	3.9 (2.2–7.1)	<0.001
Small ruminants						
Abortion	No	700	45	6.4 (4.8–8.5)	Ref.	
	Yes	325	56	17.2 (13.5–21.7)	3.0 (2.0–4.6)	<0.001
RFM	No	998	88	8.8 (7.2–10.7)	Ref.	
	Yes	27	13	48.1 (30.1–66.7)	9.6 (4.4–21.1)	<0.001
Stillbirth	No	971	87	8.9 (7.3–10.9)	Ref.	
	Yes	54	14	25.9 (15.9–32.3)	3.5 (1.9–6.8)	<0.001
Cattle						
Abortion	No	290	14	4.83 (2.87–8.0)	Ref.	
	Yes	15	7	46.67 (23.4–71.5)	17.2 (5.5–54.4)	<0.001
RFM	No	297	18	6.1 (3.8–9.4)	Ref.	
	Yes	8	3	37.5 (11.45–73.57)	9.3 (2.1–42.0)	0.004
Stillbirth	No	293	18	6.14 (3.9–9.5)	Ref.	
	Yes	12	3	25.0 (7.8–56.7)	5.1 (1.27–20.5)	0.022
Camel						
Abortion	No	239	14	5.9 (3.5–9.7)	Ref.	
	Yes	33	2	6.1 (1.5–21.7)	1.04 (0.2–4.9)	0.964
RFM	No	261	15	5.7 (3.5–9.3)	Ref.	
	Yes	11	1	9.1 (1.13–46.5)	1.6 (0.2–13.8)	0.648
Stillbirth	No	261	14	5.4 (3.2–8.9)	Ref.	
	Yes	11	2	18.2 (4.2–52.7)	3.9 (0.7–20.4)	0.104

CI, confidence interval; OR, odds ratio; RFM, retained fetal membranes.

**Table 4:** Univariable and multivariable mixed effects logistic regression model for putative risk factors for seropositivity to brucellosis in livestock species in Afar.

Factor	Category	No. Examined	No. Positive	Seropositivity % (95% CI)	Univariable OR (95% CI)	*p*-Value	Multivariable OR (95% CI)	Adjusted *p*-Value
District	Dubti	1070	56	5.2 (4.0–6.7)	Ref.			
	Amibara	1063	115	10.8 (9.1–12.8)	2.2 (1.6–3.1)	<0.001	2.1 (1.4–3.2)	<0.001
Species	Sheep	434	13	3.0 (1.7–5.1)	Ref.			
	Goats	844	105	12.4 (10.4–14.8)	4.6 (2.5–8.3)	<0.001	4.4 (2.4–8.0)	<0.001
	Cattle	429	28	6.5 (4.5–9.3)	2.3 (1.1–4.4)	0.017	2.2 (1.1–4.4)	0.023
	Camel	426	25	5.9 (4.0–8.5)	2.0 (1.02–4.0)	0.044	2.2 (1.1–4.6)	0.021
Age	Young	470	21	4.5 (2.9–6.7)	Ref.			
	Adult	1663	150	9.0 (7.7–10.5)	2.11 (1.3–3.4)	0.002	1.8 (1.1–2.9)	0.018
Sex	Female	2005	159	7.9 (6.82–9.20)	Ref.			
	Male	128	12	9.4 (3.4–15.8)	1.2 (0.6–2.2)	0.560	-	
Parity	Uniparous	324	23	7.1 (4.7–10.5)	Ref.			
	Multiparous	1280	117	9.1 (7.7–10.8)	1.3 (0.8–2.1)	0.246	-	
Herd size	Small (≤20)	865	37	4.3 (3.1–5.8)	Ref.			
	Large (>20)	1268	134	10.6 (9.0–12.4)	2.6 (1.8–3.8)	<0.001	1.9 (1.3–3.0)	0.002
Owner’s education level	None	1475	128	8.7 (7.3–10.2)	Ref.			
	Primary	658	43	6.5 (4.9–8.7)	0.7 (0.5–1.1)	0.093	-	
						Constant	0.004 (0.001–0.01)	<0.001
						Random effects	Estimate (std. err)	95% CI
						Village ID	0.01(0.06)	<0.001–23.6
						HH ID	0.23 (0.16)	0.08–0.88

CI, confidence interval; OR, odds ratio; Ref., reference; HH, household; ID, identity; std. err, standard err.

**Table 5:** Univariable and multivariable mixed effects logistic regression analysis of putative risk factors for small ruminant seropositivity to *Brucella* spp Afar, Ethiopia.

Factor	Category	No. Examined	No. Positive	Seropositivity (%)	Univariable OR (95% CI)	*p*-Value	Multivariable OR (95% CI) *	Adjusted *p*-Value
District	Dubti	641	32	5.0 (3.5–7.0)	Ref.			
	Amibara	637	86	13.5 (11.1–16.4)	3.0 (1.9–4.5)	<0.001	3.0 (1.5–5.8)	<0.001
Species	Sheep	434	13	3.0 (1.7–5.1)	Ref.			
	Goats	844	105	12.4 (10.4–14.8)	4.6 (2.5–8.3)	<0.001	4.3 (2.3–7.9)	<0.001
Age	Young (<2 tears)	208	9	4.3 (2.3–8.1)	Ref.			
	Adult (≥2 years)	1070	109	10.2 (8.5–12.2)	2.5 (1.25–5.03)	0.010	2.6 (1.2–5.7)	0.018
Sex	Female	1195	109	9.1 (7.6–10.9)	Ref.			
	Male	83	9	10.8 (5.7–19.6)	1.2 (0.59–2.49)	0.601	-	
Parity	Uniparous	201	19	9.4 (6.10–14.4)	Ref.		-	
	Multiparous	824	82	9.9 (8.1–12.2)	1.1 (0.63–1.79)	0.832	-	
Herd size	Small (≤20)	432	16	3.7 (2.3–6.0)	Ref.			
	Large (>20)	846	102	12.1 (10.0–14.4)	3.6 (2.1–6.12)	<0.001	2.6 (1.4–4.6)	0.001
Owner’s education	None	890	91	10.2 (8.4–12.4)	Ref.		-	
	Primary	388	27	6.9 (4.8–10.0)	0.7 (0.42–1.03)	0.065	-	
						Constant	0.001 (0.0002- 0.006)	<0.001
						Random-effects	Estimate (std. err)	(95% CI)
						Village ID	0.35 (0.25)	(0.09–1.43)
						HH ID	0.1 (0.15)	(0.006–1.75)

* CI, confidence interval; OR, odds ratio; Ref., reference; HH, household; ID, identity; std. err, standard error.

**Table 6:** Univariable and multivariable mixed effect logistic regression model for putative risk factors for cattle and camel *Brucella* seropositivity in two districts of Afar.

Factor	Category	No. Examined	No. Positive	Sero-Prevalence (95% CI)	Univariable OR (95% CI)	*p*-Value	Multivariable OR (95% CI)	Adjusted *p*-Value
Bovine								
District	Dubti	216	17	7.9 (4.3–11.5)	Ref.			
	Amibara	213	11	5.2 (2.3–8.1)	0.6 (0.3–1.4)	0.260	1.70 (0.67–4.31)	0.260
Age	Young (<4 years)	114	5	4.4 (0.6–8.2)	Ref.			
	Adult (≥4 years)	315	23	7.3 (4.4–10.2)	1.7 (0.6–4.6)	0.285	1.98 (0.48–8.12)	0.342
Sex	Female	398	25	6.3 (3.9–8.7)	Ref.			
	Male	31	3	9.7 (2.0–25.7)	1.6 (0.4–4.6)	0.465		
Parity	Uniparous	76	3	3.9 (0.8–11.1)	Ref.			
	Multiparous	229	18	7.9 (4.3–11.4)	2.1 (0.6–7.2)	0.252		
Herd size	Small (≤20)	181	10	5.5 (2.3–8.9)	Ref.			
	Large (>20)	248	18	7.2 (4.0–10.5)	1.3 (0.6–3.0)	0.474		
Origin	Homebred	423	26	6.1 (3.8–8.4)	Ref.			
	Acquired	6	2	33.3 (4.2–77.7)	7.6 (1.3–43.6)	0.02	6.2 (1.01–38.2)	0.049
						Constant	0.008 (0.001–0.12)	<0.001
						Random effects	Estimate (Std err)	95% CI
						Village ID	0.000	0
						HH ID	0.03 (0.72)	2.35 × 10^−24^–3.25 × 10^20^
Camel								
District	Dubti	213	7	3.2 (0.9–5.7)	Ref.			
	Amibara	213	18	8.4 (4.7–12.2)	2.7 (1.1–6.6)	0.029	3.6 (1.3–10.4)	0.017
Age	Young (<4 years)	148	7	4.7 (1.3–8.2)	Ref.			
	Adult (≥4 years)	278	18	6.5 (3.6–9.4)	1.4 (0.6–3.4)	0.467		
Parity	Uniparous	47	1	2.1 (0.05–11.3)	Ref.			
	Multiparous	227	17	7.5 (4.0–10.9)	3.7 (0.5–28.7)	0.207		
Herd size	Small (≤20)	251	11	4.4 (1.8–6.9)	Ref.			
	Large (>20)	175	14	8.0 (3.9–12.0)	1.9 (0.8–4.3)	0.117	2.02 (0.86–4.75)	0.105
Origin	Homebred	411	23	5.6 (3.3–7.8)	Ref.			
	Acquired	15	2	13.3 (1.6–40.4)	2.6 (0.5–12.2)	0.227	7.1 (1.39–35.93)	0.040
						Constant	0.2 (0.04–0.95)	0.044
						Random effects	Estimate (Std err)	95% CI
						Village ID	0.000	0
						HH ID	0.43 (0.86)	0.2 (0.01–21.9)

CI, confidence interval; OR, odds ratio; Ref., reference; HH, household; ID, identity; std. err, standard error.

## Data Availability

The data generated will be provided upon a reasonable request from the corresponding author.

## Supplementary Materials

The following supporting information can be downloaded at https://www.mdpi.com/article/10.3390/vetsci11120620/s1, Table S1: Data collection format from individual animals at blood collection, Table S2: Individual and household-level variables and their categories used during data analysis, Table S3: Mean HH livestock holdings of the sampled HHs in Amibara and Dubti districts of Afar, Ethiopia, Table S4: Household (HH) level sero-prevalence in different livestock species by district, Table S5: Village and household (HH) level sero-prevalence of brucellosis in districts of Afar and Table S6: Univariable and multivariable mixed effects logistic regression analysis for household level seropositivity to brucellosis in livestock.
