# Supplementary material for: Brucella Seropositivity and Associated Risk Factors in Pastoral Livestock System in Northeastern Ethiopia
Source: Vet Sci. 2024 Dec 3;11(12):620. doi: 10.3390/vetsci11120620 (PMC11680144; doi:10.3390/vetsci11120620)
Supplement: Supplementary file 1 [file vetsci-11-00620-s001.zip › Supplementary Table 1.pdf]

Supplementary Table S1. Data collection format from individual animals at blood collection

INDIVIDUAL ANIMAL DATA RECORDING FORMAT

Household (herd) No. _______________ Name of the owner ______________________________________ District ___________________________

Village __________________

Animal ID	Livestock species	Sex	Age	Parity	Lactation status	Lactation stage (months)	Pregnancy	Stillbirth (ever)	Abortion (ever)	Abortion over the last 3 years	RFM (ever)	Hygroma	Swollen testicles	Vaginal discharge	Origin (0= homebred, 1= introduced)
