# Supplementary material for: Brucella Seropositivity and Associated Risk Factors in Pastoral Livestock System in Northeastern Ethiopia
Source: Vet Sci. 2024 Dec 3;11(12):620. doi: 10.3390/vetsci11120620 (PMC11680144; doi:10.3390/vetsci11120620)
Supplement: Supplementary file 1 [file vetsci-11-00620-s001.zip › Supplementary Table 2.pdf]

Supplementary Table S2. Individual and household level variables and their categories used during data analysis

Variable	Variable category
District	Amibara/Dubti
Livestock Species	Goats/sheep/cattle/camel
Age	Young (<2 years for small ruminants, <4years for cattle and camels)/ Adult (>=2years for small ruminants, >=4 years for cattle and camels)
Sex	Female/ Male
Parity	Uniparous/ Multiparous
Herd size	Small (< or = 20 for each species)/ Large (>20 for each species)
Origin	Homebred/acquired
Owner's age	Young adult (<=35 years)/ Adult (>35 years)
Owner's gender	Female/male
Owner's education	None/primary
Reproductive disorders (abortion, retained fetal membranes, stillbirth)	Present/absent
Pasture ownership	Private/ communal
Water source ownership	Private/communal
Livestock contact at the household level	Yes/No
Contact at pasture	Yes/No
Contact at watering points	Yes/No
Contact with wildlife	Yes/No
Livestock breeding method	AI/Uncontrolled natural mating /controlled natural mating with known sire
Presence of parturition pen at HH level	Yes/No
Parturition occurs at	Separate parturition pen/common enclosure/pasture
Parturition waste disposal	Throw away in the field/burry/burn/fed to a dog
Management of aborting animals	Kept isolated/ kept together with the herd
