# Supplementary material for: Brucella Seropositivity and Associated Risk Factors in Pastoral Livestock System in Northeastern Ethiopia
Source: Vet Sci. 2024 Dec 3;11(12):620. doi: 10.3390/vetsci11120620 (PMC11680144; doi:10.3390/vetsci11120620)
Supplement: Supplementary file 1 [file vetsci-11-00620-s001.zip › Supplementary Table 3.pdf]

Supplementary Table S3. Mean HH livestock holdings of the sampled HHs in Amibara and Dubti districts of Afar, Ethiopia

Livestock	Dubti (No. HHs = 78)		Amibara (No. HHs = 71)	
	Mean ± Std. Err	No. HHs keeping large herd/flock	Mean ± Std. Err	No. HHs keeping large herd/flock
Goats	39.3 ± 3.0	54	50.5 ± 2.6*	69
Sheep	24.4 ± 1.6	27	30.7 ± 1.6*	41
Cattle	20.4 ± 1.4	35	24.8 ± 1.2*	45
Camel	22.0 ± 1.4	31	23.8 ± 1.4	35

HH, household; Std. Err., standard error; *significantly higher at $p<0.05$
