# Supplementary material for: Brucella Seropositivity and Associated Risk Factors in Pastoral Livestock System in Northeastern Ethiopia
Source: Vet Sci. 2024 Dec 3;11(12):620. doi: 10.3390/vetsci11120620 (PMC11680144; doi:10.3390/vetsci11120620)
Supplement: Supplementary file 1 [file vetsci-11-00620-s001.zip › Supplementary Table 4.pdf]

Supplementary Table S4. Household (HH) level sero-prevalence in different livestock species by district

| Livestock | District | No HH keeping specific livestock species | HH with seropositive livestock * | Prevalence (95% CI) |
|---|---|---|---|---|
| Goats | Amibara | 71 | 44 | 63.4 (51.1-74.5) |
| | Dubti | 70 | 21 | 30.0 (19.6-42.1) |
| | **Overall** | **141** | **65** | **46.1 (37.9-54.4)** |
| Sheep | Amibara | 71 | 11 | 16.9 (9.0-27.7) |
| | Dubti | 74 | 2 | 2.7 (0.3-9.4) |
| | **Overall** | **145** | **13** | **10.0 (4.9-14.8)** |
| Cattle | Amibara | 71 | 11 | 15.5 (8.0-26.0) |
| | Dubti | 72 | 15 | 20.8 (12.1-32.0) |
| | **Overall** | **143** | **26** | **18.2 (12.2-25.5)** |
| Camel | Amibara | 71 | 16 | 22.5 (13.5-34.0) |
| | Dubti | 71 | 6 | 8.4 (3.2-17.5) |
| | **Overall** | **142** | **22** | **15.5 (10.0-22.5)** |
| **All species** | | **149** | **89** | **59.7 (51.6-67.4)** |

*some households had multiple seropositive livestock species. CI: confidence interval
