# Supplementary material for: Brucella Seropositivity and Associated Risk Factors in Pastoral Livestock System in Northeastern Ethiopia
Source: Vet Sci. 2024 Dec 3;11(12):620. doi: 10.3390/vetsci11120620 (PMC11680144; doi:10.3390/vetsci11120620)
Supplement: Supplementary file 1 [file vetsci-11-00620-s001.zip › Supplementary Table 5.pdf]

Supplementary Table S5. Village and household (HH) level sero-prevalence of brucellosis in districts of Afar

**District**	**Village**	**Individual animal-level *Brucella* seropositivity**			**HH-level animal brucellosis sero-prevalence**		
		No. sampled	No. positive	Prevalence (95% CI)	No. HH sampled	No. HH positive	Prevalence (95% CI)
Amibara	Village 1	297	34	11.4 (8.3-9.2)[a]	20	15	75.0 (51.3–89.5)
	Village 2	333	39	11.7 (8.7-15.6)[a]	22	17	77.3 (54.8–90.5)
	Village 3	255	21	8.2 (5.4-12.3)[a,b]	17	12	70.6 (44.8–87.6)
	Village 4	178	21	11.8 (7.8-17.4)[a]	12	9	75.0 (43.1–92.2)
Dubti	Village 5	261	10	3.8 (2.1-7.0)[b]	18	7	38.9 (19.2–63.0)
	Village 6	174	16	9.2 (5.7-14.5)[a,b]	12	8	66.7 (36.1–87.6)
	Village 7	308	11	3.6 (2.0-6.3)[b]	21	10	47.6 (27.3–68.8)
	Village 8	171	15	8.8 (5.3-14.1)[a,b]	14	8	57.1 (30.6–80.1)
	Village 9	156	4	2.6 (1.0-6.6)[b]	13	3	23.1 (7.2–53.7)

[a,b]rows with different subscripts are significantly different ($p<0.05$), CI, confidence interval
