# Supplementary material for: Brucella Seropositivity and Associated Risk Factors in Pastoral Livestock System in Northeastern Ethiopia
Source: Vet Sci. 2024 Dec 3;11(12):620. doi: 10.3390/vetsci11120620 (PMC11680144; doi:10.3390/vetsci11120620)
Supplement: Supplementary file 1 [file vetsci-11-00620-s001.zip › Supplementary Table 6.pdf]

Supplementary Table S6. Univariable and multivariable mixed effects logistic regression analysis for household level seropositivity to brucellosis in livestock.

Factor	Category	No. examined	No. positive	Sero-prevalence	OR (95% CI)	p-value	Multivariable OR (95% CI)	*p-value
District	Dubti	78	36	46.1 (35.3-57.4)	Reference			
	Amibara	71	53	74.6 (63.1-83.5)	3.3 (1.6-6.5)	0.001	3.7 (1.8 -7.7)	<0.001
Owner's Age	<=35 years	47	23	48.9 (34.4-63.5)	Ref.			
	>35 years	102	66	64.7 (55.3-74.1)	1.9 (0.9-3.7)	0.07	2.2 (0.9-5.6)	0.079
Owner's gender	Female	17	11	64.7 (38.2-85.8)	Ref			
	Male	132	78	59.8 (51.4-68.3)	0.79 (0.27-2.51)	0.657		
Education	None	102	64	63.7 (54.3-73.2)	Ref			
	Primary	47	25	51.1 (36.5-65.6)	0.59 (0.29-1.20)	0.270		

OR, odds ratio; CI, confidence interval, *p-value, adjusted p-value from the multivariable model
